# Effect of *Huntiella decorticans* and drought on *Nothofagus dombeyi* seedlings

**DOI:** 10.1093/aobpla/plad068

**Published:** 2023-10-10

**Authors:** Pablo Masera, María Belén Pildain, Mariano Aquino, Andrés De Errasti, Guillermina Dalla Salda, Mario Rajchenberg, María Florencia Urretavizcaya

**Affiliations:** Centro de Investigación y Extensión Forestal Andino Patagónico (CIEFAP), Ruta 259 Km 16.24, CC14 (9200), Argentina; Consejo Nacional de Investigaciones Científicas y Técnicas (CONICET), (1425)Argentina; Centro de Investigación y Extensión Forestal Andino Patagónico (CIEFAP), Ruta 259 Km 16.24, CC14 (9200), Argentina; Consejo Nacional de Investigaciones Científicas y Técnicas (CONICET), (1425)Argentina; Centro de Investigación y Extensión Forestal Andino Patagónico (CIEFAP), Ruta 259 Km 16.24, CC14 (9200), Argentina; Centro de Investigación y Extensión Forestal Andino Patagónico (CIEFAP), Ruta 259 Km 16.24, CC14 (9200), Argentina; Consejo Nacional de Investigaciones Científicas y Técnicas (CONICET), (1425)Argentina; Consejo Nacional de Investigaciones Científicas y Técnicas (CONICET), (1425)Argentina; Instituto Nacional de Tecnología Agropecuaria (INTA), Grupo de Ecologia Forestal, (8400)Argentina; Centro de Investigación y Extensión Forestal Andino Patagónico (CIEFAP), Ruta 259 Km 16.24, CC14 (9200), Argentina; Consejo Nacional de Investigaciones Científicas y Técnicas (CONICET), (1425)Argentina; Centro de Investigación y Extensión Forestal Andino Patagónico (CIEFAP), Ruta 259 Km 16.24, CC14 (9200), Argentina; Consejo Nacional de Investigaciones Científicas y Técnicas (CONICET), (1425)Argentina

**Keywords:** Ecophysiology of Patagonian forest species, living wood pathogens, ophiostomatoid fungi, multiple stressors

## Abstract

In the temperate forests of Patagonia, Argentina, *Nothofagus dombeyi*, commonly known as Coihue, has shown sensitivity to intense drought events, leading to mortality. Studies have been conducted on Coihue decline and mortality using a variety of approaches, including the role of extreme heat waves and drought, pests and pathogens, particularly the fungus *Huntiella decorticans*. This work aimed to evaluate survival, vitality, necrosis extension and growth response of inoculated and non-inoculated Coihue seedlings from different provenances exposed to different soil moisture levels. To achieve this goal, 96 Coihue seedlings from 2 different provenances were assigned to 8 different experimental treatments. Treatments were composed of the presence or absence of *H. decorticans* and different soil moisture content conditions, dry, wet and the exposure to dry condition at different times of the experiment. Both dry conditions and *H. decorticans* had negative effects on the survival and growth rate of Coihue. The combination of both factors resulted in 100 % mortality, regardless of the plants’ geographical provenances. Mortality began to be observed 3 months after pathogen inoculation, during the warmest month. Necrosis extension produced by the pathogen was similar for most of the inoculated treatments. The treatment under wet condition during the experiment but subjected to dry condition in the previous growing season presented lower necrosis extension (8.4 ± 3.2 %), than the other treatments. The radial increase was also affected by the treatments and geographical provenance, being those plants exposed to dry conditions which grew less (0.19 ± 0.21 mm). This study enhances our understanding of the plant–pathogen interaction. According to our results, Coihue may become more susceptible to mortality, when *H. decorticans* and water deficit conditions act synergistically.

## Teaser

Coihue trees (*Nothofagus dombeyi* ), icons of Patagonia’s ancient forests, face challenges from droughts and fungal diseases. Nursery-controlled experiments revealed that droughts left Coihue seedlings vulnerable to lethal fungal infection by *Huntiella decorticans* . Nevertheless, prior drought exposure also activated the trees’ defenses, reducing the fungus’ impacts. Looking ahead, climate shifts could lead to more droughts in the future, which would allow this endemic forest pathogen to become more critical.

## Introduction

Wild populations and their habitats are exposed to numerous stressors that can disrupt their normal functioning (Geldmann *et al.* 2014). These stressors can interact in complex ways, and the combined effect of two or more of these factors can be synergistic or antagonistic, depending on their individual effects (Folt *et al.* 1999). Particularly, droughts could alter vegetation and modify their interactions with biotic agents (Sangüesa-Barreda *et al.* 2015). Low water potential in tissues during a dry period limits plant cell metabolism by reducing the amount of water available for these processes. This negatively influences carbohydrate production and translocation, as well as the production of resins and other metabolites necessary for plant defense against biological attacks (Hammerschmidt and Schultz 1996). This means that physiological processes that predispose plants to mortality are associated with limited cell metabolism (Choat *et al.* 2018). Considering that the Intergovernmental Panel on Climate Change report in 2022 indicates that the current global temperature is 1.5 °C higher than in pre-industrial times, an increase in the frequency and intensity of droughts would be expected. In addition, temperature increment in a climate change context could create favourable conditions for the development of harmful agents (Sturrock *et al.* 2011). Examples of this type of disease are *Phytophthora* root rot (Bergot *et al.* 2004) or decline disease like acute oak decline (Brown *et al.* 2016). This severely affects forest productivity and may cause loss of biodiversity (Millar and Stephenson 2015).

**Figure 1.**
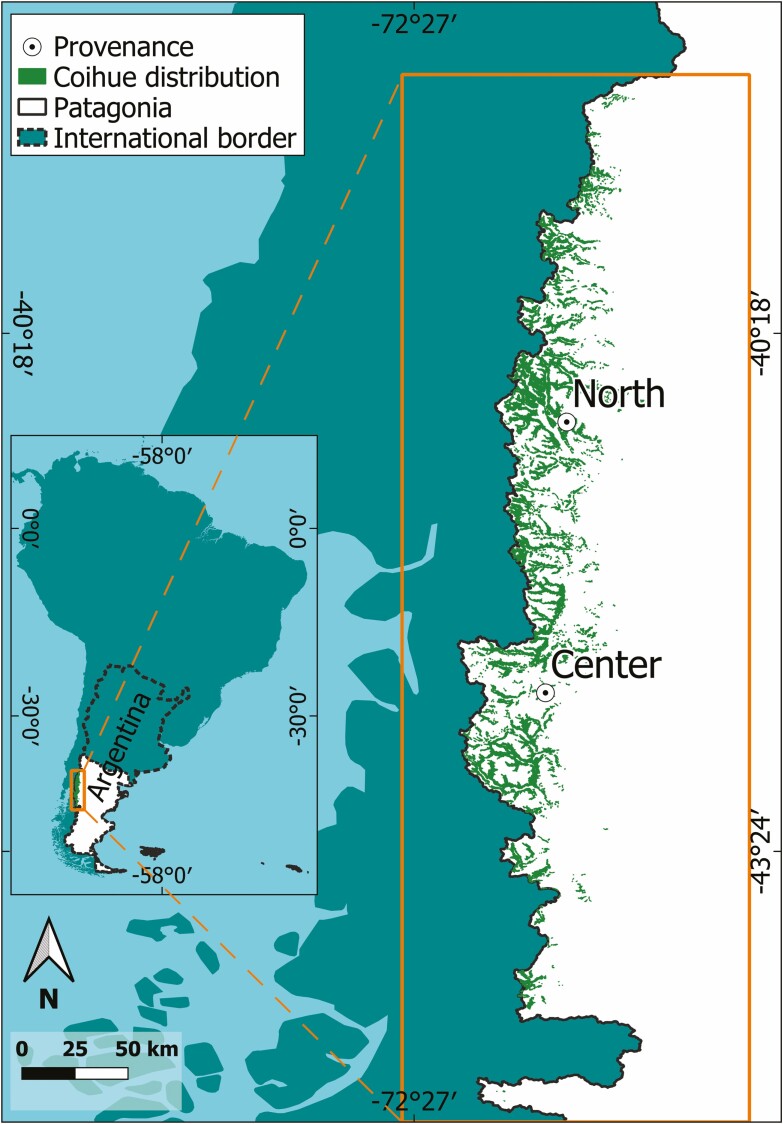
Coihue distribution in Patagonia (CIEFAP-MAyDS 2016) and origin of the seeds to produce the seedlings used in this work.

In Patagonia Argentina, distribution of *Nothofagus dombeyi*, commonly known as Coihue, occurs from the centre-west zone of Neuquén province to the central region of Chubut province (Fig. 1). Coihue is one of the most abundant native species of the Andean Patagonian forests, becoming dominant in many mixed forests or forming pure forests (Donoso *et al.* 2006). Among the six endemic species of the genus *Nothofagus* that inhabit Argentine Patagonia, this evergreen tree is the most restricted by humidity conditions (Bisheimer and Fernández 2000; Donoso *et al.* 2006), and possibly, the most susceptible to drought. Thus, the changes in climatic trends observed in the western part of the Andes Mountain range, as the annual average rainfall and water flow decrease (Ludeña *et al.* 2012), could directly affect Coihue forest dynamics (Suarez and Kitzberger 2008). Although it would be expected that Coihue would present structural and functional intraspecific variability with a genetic basis due to its wide range of distribution and the variation in the physical environment, little is known about the possible adaptations of the species to different environmental scenarios, due to the lack of studies on the matter (Premoli *et al.* 2012).

With different approaches, studies have been conducted on Coihue episodic mortality caused by extreme heat waves and drought (Suarez *et al.* 2015; Molowny-Horas *et al.* 2017), pests (FAO-CONAF 2008) and by fungal pathogens (de Errasti *et al.* 2015; Molina *et al.* 2020). Related to pathogens in native Coihue forest, research has been done on fungi of the *Ceratocystidaceae* (Ascomycota) family, which includes saprophytic (De Beer *et al.* 2013) and pathogenic organisms of Fagales from different genera. These pathogens cause typical vascular wilts (Henry 1944) and vascular stains (Kile and Walker 1987). In 2015, a group of isolates from diseased Coihues in Patagonia was described as *Huntiella decorticans* (de Errasti *et al.* 2015). However, these isolates were able to colonize healthy living trees and cause disease in pathogenicity trials. Moreover, ‘pressure pad’ formation (Morris and Fergus 1952) and its association with nitidulid or sap-feeding beetles suggest that *H. decorticans* infects healthy trees in nature, like *Bretziella fagacearum* (Cease and Juzwik 2001). The goals of this work were to evaluate the response of Coihue seedlings from different geographical provenances to different soil moisture levels and the presence of *H. decorticans*, under experimental conditions. We postulated that *H. decorticans* present in forests of this species behave as pathogens with the ability to cause disease, under drought conditions, causing mortality in young individuals. Based on this, we suggest that low soil moisture content could predispose Coihue seedlings to suffer disease and death, caused by *H. decorticans*, as a synergistic stress effect. Also, considering that little is known about the genetic and phenotypic variability among Coihue populations and their adaptive capacity, we aimed to address the following question: can Coihue geographical provenances from different latitudes influence plant growth and show different behaviour against the pathogen and different soil moisture contents?

## Materials and Methods

### Plant material and conditioning

The study was carried out at the Andean Patagonian Forest Research and Extension Center (CIEFAP) (42°55ʹ50.64″S, 71°21ʹ49.95″W), in Esquel city, Chubut Province, Argentina, under semi-controlled conditions. Coihue seedlings were obtained from two nurseries in the region: one in Bariloche city, Río Negro Province, and another in Esquel, Chubut Province. The selection of these two sites was made for two reasons. In the first place, there are not many producer nurseries with the availability of seedlings of the species, and on the second place, the seedlings were produced with seeds from distant populations at different latitudes. Seedlings from Bariloche (northern geographical provenance) were grown from seeds collected in Nahuel Huapi National Park (40°49ʹ50.67″S, 71°32ʹ14.00″W), while Esquel seedlings (central geographical provenance) were grown from seeds collected in the Cholila lake area (42°27ʹ4.57″S, 71°39ʹ51.76″W). We used 2-year-old seedlings from the northern area, which were produced as bare-root plants in open-air seedbeds, and 1-year-old seedlings from the central area, which were cultivated in containers in a covered-root system.

In September 2019 (early spring in the Southern Hemisphere), we transplanted 96 seedlings from each geographical provenance into 3.5-L plastic containers, with substrate composed of two parts common topsoil from the area and one-part volcanic sand. To reduce transplant stress, we applied foliar fertilizer (Nuquifol Premium brand), composed of nitrogen (total 8.5 %), phosphorus (assimilable 4.5 %) and potassium (soluble 7 %). To aid their establishment, we placed the plants in partial shade with 50 % coverage and normal irrigation for 2 weeks. We selected 12 plants from each provenance to expose them to dry condition (30–50 % field capacity [FC]) during the growing season of 2019–20, which was the year prior to setting up the experiment. The remainder were maintained with periodic irrigation up to FC.

In early spring 2020, we transplanted all plants to 5-L containers with the same substrate as described above, and we applied the same foliar fertilizer. We irrigated all plants to saturation and allowed them to drain before being weighed to determine the level of FC soil moisture. For 2 weeks plants were kept under regular irrigation sheltered in roofed structures with polycarbonate plates with a light transmission level 50 % (Carbolux brand), to facilitate their establishment.

### Soil moisture conditions

We defined two soil moisture content conditions for the experiment: dry (30–50 % FC) and wet (70–90 % FC). These moisture content conditions were maintained throughout the experiment by manual watering. We assume that at this level of dry conditions, the effects of drought begin on the Coihue seedlings. This is based on the studies of Caselli *et al.* (2019), where it was determined through a nursery trial that 20 % of the soil moisture content of the FC is the lowest moisture limit for the survival of Coihue seedlings.

To check the water status of the soil and to define the amount of water to be added during the experiment, we selected two plants per provenance per treatment 32 in total to measure their substrate moisture content gravimetrically once a week, during the experiment (Caselli *et al.* 2019). Previously, a sample of the substrate used for the plants had been watered to saturation, drained for 24 h, and weighed. The sample was then placed in a drying oven for 48 h at 103 ± 2 °C and weighed again to obtain dry weight. Finally, the amount of water corresponding to FC of the substrate was calculated according to Equation (1) (Shukla *et al.* 2014).


$$\begin{array}{l} water\;content\left(\;\%\;\right)=\frac{\left(wet\;weight-dry\;weight\right)}{wet\;weight}\times 100. \end{array}$$

Equation 1: gravimetric water content based on the difference in wet and dry weight of the substrate. Where the wet weight of the substrate is obtained after water saturation and subsequent drainage; and the dry weight is obtained after drying the substrate for 48 h at 103 ± 2 °C.

### Experimental design

At the beginning of spring 2020, we set a two-factor experiment. The first factor was provenance, with two levels. The second factor was treatment, with eight experimental levels (Table 1) of different combinations of soil moisture content and *H. decorticans* inoculation. Each provenance treatment combination had 6 replicated seedlings (1 per pot), with a total of 12 replicates per treatment. The first four treatments corresponded to a factorial combination of dry versus wet and an arrangement of two conditions of moisture content and inoculation with *H. decorticans* (CIEFAPcc 452) (from de Errasti *et al.* 2015; Molina *et al.* 2020) or not. The soil moisture content condition was defined as 40 % of FC (dry) and 80 % of FC (wet or control). The pathogen condition was inoculated (P: agar + *H. decorticans*) or not inoculated (agar). To better understand the effects of moisture variations at different times, related to interannual drought events in Patagonia and to evaluate specifically the interaction effects of this variation with *H. decorticans*, two additional treatments were established, in which the moisture content condition was inverted after inoculation (Hossain *et al.* 2020; van Kampen *et al.* 2022). In the pre-inoculation dry condition plant level, the moisture condition was switched to wet condition after inoculation (dry->Pwet); conversely, the level that had been subjected to wet condition pre-inoculation was switched to dry condition after inoculation (wet->Pdry), this applied to both provenances. Finally, two additional treatments were subjected to dry condition during the 2019–20 growing season (spring-summer) (yr1dry), to evaluate whether weakening caused by low soil moisture content in previous seasons increased susceptibility to disease caused by the pathogen. The seedlings in both treatments were inoculated and exposed to dry condition (yr1dry->Pdry) and wet condition (yr1dry->Pwet), respectively (Table 1). All plants were kept under the same polycarbonate roof structures mentioned above until the end of the experiment. In total, 96 plants were used for the experiment, and a 10-plant sample per provenance for morphological characterization. The experiment was concluded after 8 months, until the end of April 2021, coinciding with the end of the growing season.

**Table 1.** Description of the eight treatments representing different combinations of moisture content and pathogen inoculation.

Treatment	Description
wet	No inoculation, soil moisture constant at 80 % of field capacity.
dry	No inoculation, soil moisture constant at 40 % of field capacity.
Pwet	Pathogen + soil moisture constant at 80 % of field capacity.
Pdry	Pathogen + soil moisture constant at 40 % of field capacity.
dry->Pwet	Pathogen + initial soil moisture at 40 % of field capacity then switched to soil moisture at 80 % of field capacity.
wet->Pdry	Pathogen + initial soil moisture at 80 % of field capacity then switched to soil moisture at 40 % of field capacity.
yr1dry->Pwet	Soil moisture at 40 % of field capacity legacy + pathogen + soil moisture constant at 80 % of field capacity.
yr1dry->Pdry	Soil moisture at 40 % of field capacity legacy + pathogen + soil moisture constant at 40 % of field capacity.

To measure soil temperature during the experiment, we installed a data logger (Decagon Em5b) and two sensors. One sensor was located within a witness plant container in wet treatment and another in dry treatment at 5 cm substrate depth. Data were recorded every half hour during the experiment. The soil temperature of the wet and dry treatments was compared by a *t*-test. We found differences in soil temperature between both moisture content (dry and wet) (*P* < 0.001), being the greater temperature recorded in the lower soil moisture. During the study period, monthly average temperature was higher (*P* < 0.001) in December, January and February, being the warmest months of the growing season (Supplementary material 1).

### Fungal isolate and inoculation

For this experiment, we used *H. decorticans* ex-type culture CIEFAPcc 452 (CIEFAP culture collection) isolated from *N. dombeyi* by de Errasti *et al.* (2015) characterized as the most pathogenic isolates within pathogenicity trials (de Errasti *et al.* 2015; Masera *et al.* 2022). For inoculum production, CIEFAPcc 452 was grown for 4 weeks on MEA 2 % amended with 1 % Coihue sawdust (de Errasti *et al.* 2015). For pathogen inoculation, we did window-shaped incisions 2 cm above the plant stem base, to expose xylematic conductive tissue. The tools were surface sterilized after every inoculation. We deposited 0.5 cm diameter plugs with mycelium or agar on the exposed tissues, depending on the treatment. The wound was covered with autoclaved moistened gauze with distilled water and aluminium foil to conserve moisture and prevent external contamination (de Errasti *et al.* 2015). In this sense, it is worth noting that there are species of pathogenic fungi from which is difficult to obtain material such as spores or reproduce its life cycle. In these cases, like *H. decorticans*, isolation, inoculum production and artificial inoculation are suitable techniques (Bhunjun *et al.* 2021). We inoculated the plants 40 days after moisture content treatments were set, we assume a physiological effect on the seedlings as a result of the moisture content variations (Caselli *et al.* 2019; Hossain *et al.* 2020).

## Measurements

### Seedling morphological attributes

To determine if there were morphological differences among seedlings from the different nurseries and provenances and perform a morphological characterization of the initial material, we conducted destructive measurements and subsequent statistical analyses on a sample of seedlings.

Prior to the moisture condition/inoculation experiment, in September 2020, seedling samplings from each provenance (*n* = 10) were randomly chosen. We measured shoot height from the stem base to the dominant terminal bud on the upper branch (H, cm), stem base diameter at 1 cm above the cotyledon insertion point (SBD, mm) and root length (RL, cm). Shoot dry weight (SDW, g), comprising the stem and leaves, and root dry weight (RDW, g), were determined by weighing after being dried at 103 ± 2 °C for 48 h. The slenderness index (H × 10/SBD) and the shoot to root ratio were also calculated (Hasse 2007).

We compared the morphological parameters of seedlings from each provenance by a *t*-test for most of the parameters except for shoot height and SDW, where we used a Kruskal–Wallis test because normality assumption (Shapiro–Wilk test) nor variance homogeneity (Levene’s test) were not met, even after variable transformation.

Seedlings presented differences between both provenances at the beginning of the experiment (Table 2) in height, RL, RDW and slenderness index. Central provenance seedlings showed greater RL, H development and slenderness than northern provenance seedlings.

**Table 2.** Average morphological parameters and standard error at the beginning of the experiment (*n* = 20. ANOVA Df = 18, Kruskal–Wallis Df = 1).

	Centre		North		
Source	Mean	SE	Mean	SE	*P* value
Stem base diameter (mm).	7.67	0.21	6.69	0.55	0.194
Shoot height (cm)	62.16	2.07	43.94	5.63	0.023
Root length (cm)	32.70	1.68	27.25	1.52	0.027
Root dry weight (g)	7.14	0.76	3.95	0.74	0.004
Shoot dry weight (g)	9.19	0.62	6.83	1.51	0.130
Slenderness index	81.61	3.38	64.84	5.36	0.016
shoot to root ratio	1.66	0.14	1.38	0.13	0.150

### Survival, vitality, extent of necrosis and growth

For the experiment involving the eight treatments, we carried out five measurement campaigns during the 2020–21 growing season and recorded survival, vitality and growth in all plants. We determined survival by scoring individual plants as 0 not survived or 1 survived. To evaluate vitality, we used a scale of severity levels modified from Boa (2003) and Caselli *et al.* (2019). The levels were: healthy, yellowing leaves, necrotic leaves, wilted leaves and branches, wilted crown and necrosis at glance in superficial tissues. For each treatment, the percentage of plants with different levels of vitality was recorded.

During the experiment dead plants were harvested and, to confirm the infection and relate the symptoms to the inoculated pathogen, its re-isolation was carried out from necrotic tissue of each plant. At the end of the assay, all surviving plants were harvested, and the presence of the pathogen was confirmed, same as detailed for dead plants. In the inoculated plants, we measured the length of necrosis produced in the sapwood by *H. decorticans* through cross-sections of the stem from 1 to 2 cm with necrosis. The cuts were made from the point of inoculation both toward the apex of the plant and the roots. Lengths of the sections showing necrosis were added to obtain the total length of the extension. We determined with this data the percentage of the stem occupied by the lesion (de Errasti *et al.* 2015). *Huntiella decorticans* identification included morphological and DNA sequence comparison of tubulin region, according to de Errasti *et al.* (2015).

To estimate growth, we measured shoot height and stem base diameter in each campaign. We determined total growth by deducting initial values to final values, for each parameter. At the end of the experiment, partial increments were determined between campaign measures the same way as the total detailed for total growth.

### Analyses

We evaluated the effects of treatments and provenances on survival, necrosis extension and growth based on stem base diameter and height increments, by a two-way ANOVA. Stem base diameter and height total increment were studied throughout the experiment for those plants that survived until the final harvest. For survival, we performed a generalized model with a binomial error distribution, using ‘glm’ function, while for the rest of the parameters, we implemented a linear model, using the ‘lm’ function in r package (Posit Team 2023). Linear models were conducted with main effects (Equation (2)) and with their interaction (Equation (3)) to apply the ANOVA. In cases where the interactions between the independent variables were not significant, we used the main effects model.


$$\begin{array}{l} variable=\beta 0+\beta 1\left(treatment\right)+\beta 2\left(provenance\right)+\varepsilon . \end{array}$$

Equation 2: Main effects model where variable is the dependent variable, *β*0 is the constant term or intercept, *β*1 and *β*2 are the coefficients that multiply each term, treatment and provenances are the independent variables and *ε* is the random error term.


$$\begin{array}{lll} variable= & \beta 0+\beta 1\left(treatment\right)+\beta 2\left(provenance\right)\; & +\beta 3\left(treatment:provenance\right)+\varepsilon . \end{array}$$

Equation 3: Interaction model where variable is the dependent variable, *β*0 is the constant term or intercept, *β*1–*β*3 are the coefficients that multiply each term, treatment and provenances are the independent variables and the term treatment:provenances represent the interactions between the two variables. *ε* is the random error term.

Considering that all the seedlings that showed total mortality were those exposed to dry conditions, we performed a regression analysis and a two-way ANOVA to determine the differences in trend partial increases in stem base diameter before the end of the experiment, using the moisture content condition as a factor, regardless of the inoculation condition.

We used the LSD Fisher test (least-significant-difference) where significant differences were detected (*P* ≤ 0.05). Prior to the analyses, the data were transformed in cases where normality and heteroscedasticity were not met. For the statistical analysis, native R software (RStudio 2023.03.0 + 386 ‘Cherry Blossom’ Release) and agricolae package (Mendiburu 2020) functions were used.

## Results

### Survival, vitality and necrosis extension

Even though seedlings exhibited initial morphological differences among provenances (Table 1), there were neither significant effects of provenance nor the interaction between provenance and treatments for either survival or necrosis extension. There were highly significant effects of treatment for both survival and necrosis extension (Table 3). There also was no clear difference among provenances for the vitality observations. Therefore, we report below just the results for the treatment effects, pooled across the two provenances.

**Table 3.** ANOVA results for the main effects linear model on survival rate and necrosis extension.

Source	Df	Sum of squares	Mean square	*F* statistic	*P* value
Survival					
Treatment	7	19.12	2.73	52.82	<0.001
Provenance	1	0	0	0	1
Residuals	87	4.50	0.05		
Necrosis extension					
Treatment	5	17.09	3.42	5.11	<0.001
Provenance	1	0.01	0.01	0.01	0.913
Residuals	60	40.15	0.67		

**Figure 2.**
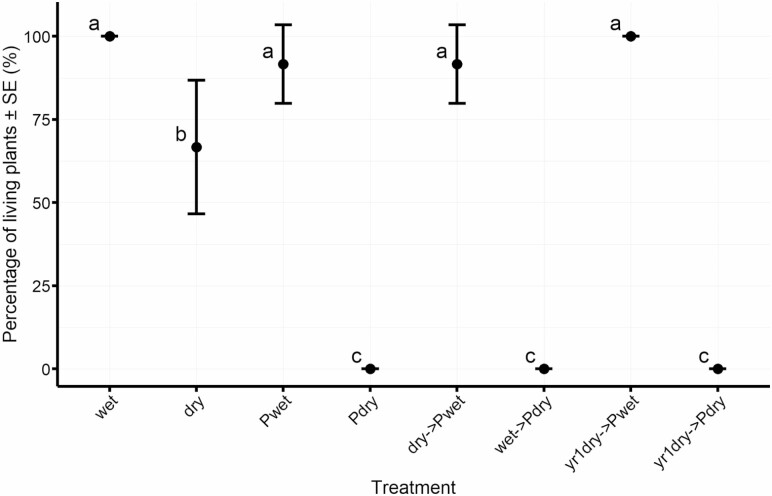
Survival presented in terms of percentage of living plants for each treatment. Treatments abbreviations as described in Table 1. Means with letters in common are not significantly different (*P* > 0.05).

Coihue mortality started 3 months after pathogen inoculation, when the highest soil temperatures were recorded. Between March and April plants inoculated with the pathogen and exposed to dry conditions during the experiment (Pdry, wet->Pdry and yr1dry->Pdry) presented abrupt and complete mortality. Contrary, those treatments exposed to wet conditions, regardless of the presence of the pathogen presented high survival (wet, Pwet, dry->Pwet and yr1dry->Pwet), while in dry treatment medium survival rate was registered (Fig. 2).

**Figure 3.**
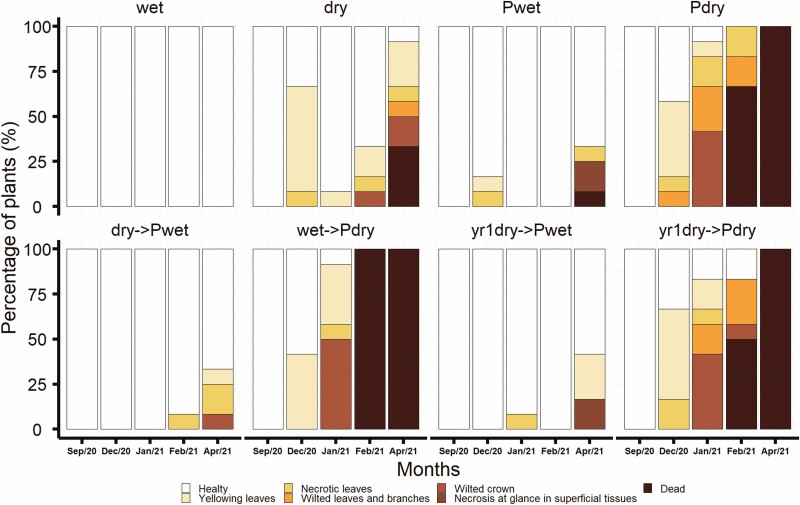
Seedling vitality presented in terms of percentage of symptomatic plants for each treatment. Treatments abbreviations as described in Table 1.

Regarding vitality, seedlings dry content treatments (dry, Pdry, dry->Pdry, yr1dry->Pdry) presented leaves discolouration to yellow and necrosis after 2 months of inoculation. These symptoms increased in severity as the experiment progressed, showing wilted branches spreading to all the crowns until the seedling died. The less affected treatments were those inoculated with *H. decorticans* and wet conditions (Pwet, dry->Pwet, yr1dry->Pwet), where we observed sporadically some yellowing leaves until the end of the experiment, when we could observe some necrosis in superficial conductive tissues (Fig. 3).

**Figure 4.**
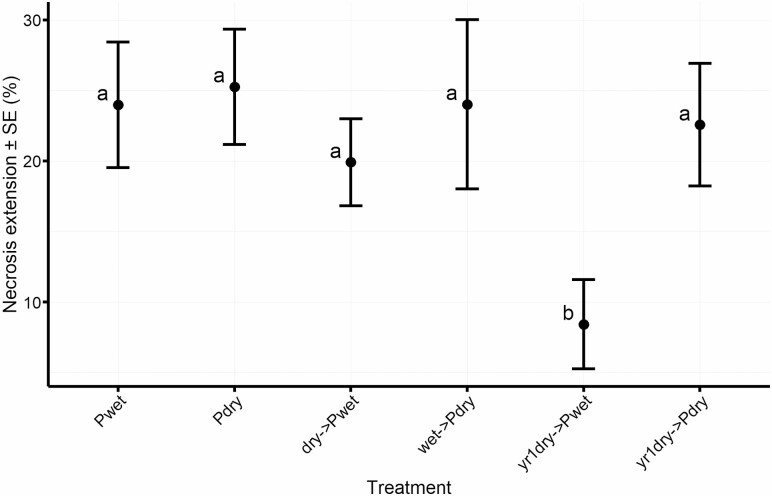
Average necrosis extension (%) in plants inoculated with *H. decorticans*. Treatments abbreviations treatments as described in Table 1. Means with letters in common are not significantly different (*P* > 0.05).

We confirmed the identity of the pathogen by reisolating *H. decorticans* from necrotic tissues, and corroborated that it was the lesion causing agent. The shortest necrosis extension we recordered (8.43 ± 3.17 %) was in the treatment inoculated with the pathogen and exposed to wet condition with a previous exposure to dry condition (yr1dry->Pwet), regardless of provenance (Fig. 4).

### Growth

We did not find significant interactions between treatment and provenance for total height parameter. Contrary, we found significant differences in the total stem diameter increase at the end of the experiment for all the variables, but no interaction between them was detected (Table 4).

**Table 4.** ANOVA results for the main effects linear model on total stem base diameter increase.

Source	Df	Sum of squares	Mean square	*F* statistic	*P* value
Stem base diameter increase					
Treatment	4	26.05	6.51	9.54	<0.001
Provenance	1	5.03	5.03	7.37	<0.001
Residuals	54	36.88	0.68		

**Figure 5.**
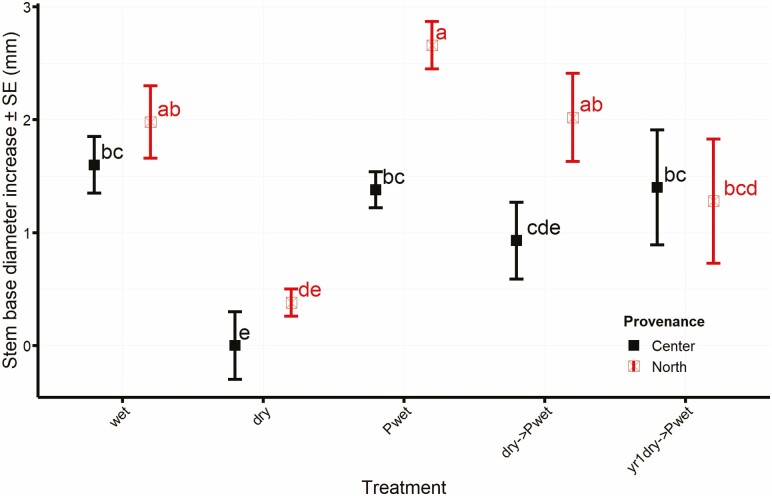
Average total increase by treatment and provenance, in treatments with surviving plants at the end of the experiment. Treatments abbreviations as described in Table 1. Means with letters in common are not significantly different (*P* > 0.05).

Centre provenance seedlings showed smaller increases than northern provenance seedlings. Treatment not inoculated and exposed to dry condition had the lowest increase in stem base diameter (Fig. 5).

Linear slope for treatments with wet conditions (wet, Pdry, yr1dry->Pwet and dry->Pwet), regardless of the provenance, were positive. In contrast, the slope of those treatments exposed to dry conditions (dry, Pdry, yr1dry->Pdry and dry->Pwet), depending on the provenance, was negative for north provenance or had a light positive slope compared with high moisture content, for centre provenance (Supplementary material 2).

## Discussion

This study addressed, under experimental conditions, the effect of the pathogenic organism *H. decorticans* on Coihue seedlings survival and growth when exposed to different levels of soil moisture content. The results indicated negative effects of both dry conditions on soil and the pathogen; however, the combination of both factors showed a much greater severity in the development of the disease, regardless of the geographical provenances. These findings suggest that drought and *H. decorticans* may be synergistic in their effects on Coihue seedlings.

*Huntiella decorticans* is a frequent pathogen in *Nothofagus* forests, associated with structures such as pressure pads and certain insects. However, our knowledge of the incidence and severity in young Coihue plants, as well as how this pathosystem could be affected by climate change, is limited. We use traditional concepts in plant pathology as the disease triangle (Gäumann 1950) and Koch’s postulates (Agrios 2005) with the inclusion of emerging ecological concepts in host–pathogen interactions, such as the role of soil moisture to focus on the roles of climate (Pautasso *et al.* 2012). Dissecting the natural ecosystem into its components: plant—pathogen—soil moisture interaction in a controlled experimental assay, allows us to understand the potential primary climate effects and its impact in *N. dombeyi*—*H. decorticans* pathosystem. This framework offers a basis to evaluate hypotheses regarding climate’s involvement in forest diseases. A foundational principle for this approach is to provide context. The disease can be replicated through controlled inoculation and conditions, which assures the presence of the pathogen allowing its evaluation and contrasting the proposed hypothesis. If pathogens are involved, inoculations under specific conditions may be useful to indicate their ability to cause harm. Often, experimentation with climate controls under natural conditions may not be feasible, but exposing seedlings to climatic conditions may indicate a pattern in the forest ecosystems (Pautasso *et al.* 2012). Especially in tree regeneration, a fundamental process in forest ecosystems ensuring the persistence and resilience of forest stands.

### Effects of provenances on seedling development during the experiment

The development of the Coihue seedlings during this experiment, based on the measured parameters, did not show differences between the two provenances used in this study, except for the stem base diameter, which is discussed further below. This may be because the populations are established in similar habitats. Regarding precipitation, although the provenances are latitudinally separated (180 km approximately), precipitation gradients in the region occur longitudinally, from west to east. In this sense, both provenances are located between the isohyets ranging from 1000 mm per year to 800 mm per year (Karger *et al.* 2017). Mean annual temperatures are similar for both places, 9 °C for centre provenance and 8 °C for north provenance (Karger *et al.* 2017). In terms of geomorphology, the landscape for both provenances is the result of the Andean orogeny, whose characteristics are the product of Quaternary glacial and volcanic activity (Matteucci 2012). This lack of difference between environmental conditions of both Coihue populations could explain the same response to the presence of this endemic pathogen.

### Effects of moisture content and inoculation on survival and vitality

The results prove that Coihue is sensitive to low moisture conditions, and this effect is even more severe when combined with fungal inoculation. In treatments exposed to low moisture content alone, moderate plant mortality was observed, whereas for treatments exposed to both dry conditions and the pathogen, no seedlings survived until the end of the experiment. Sensitivity to water deficiency has been registered and studied in Coihue forests with large-scale mortality. In these forests mainly young trees were affected (Molowny-Horas *et al.* 2017; Caselli *et al.* 2019). In this sense, mortality on seedlings observed may be due to a failure of hydraulic mechanisms and carbon balance as described for other species (Brodribb & McAdam 2011). This implies that xylem pressure drops below the cavitation threshold, interrupting water transport and causing the plant to dry out irreversibly (McDowell *et al.* 2008) and young plants are particularly susceptible to this phenomenon (Choat *et al.* 2018). Although mortality of the Coihue seedlings is a common event during a normal drought period, an intensification of these events and also a modification in the timing of occurrence could limit the canopy development in the future. This due to the reduction in the abundance of this dominant species (Suarez and Kitzberger 2008; Suarez *et al.* 2015).

Plants have physiological mechanisms that allow them to adapt to water deficit conditions. These plants can be divided into two categories according to their water regulation behaviour through stomatal closure: anisohydric plants, which do not close their stomata due to a decrease in soil water potential, and isohydric plants, which do close their stomata (Tardieu 1993; Tardieu and Simonneau 1998). Response of Coihue to periods of water deficit has been studied by Díaz *et al.* (2020), who concluded that populations growing in drier biogeographic areas present isohydric behaviour that allows some resistance to water stress. However, the moderate mortality observed in dry treatment could indicate some sensitivity to water deficit, a product of anisohydric plant behaviour regardless of provenance, and exacerbated by the young age of the plants. In addition, soil temperature can also be a stressor factor for the plant. Although not many studies have focused on thermal shock in plants of the *Nothofagus* genus, Barcala’s work (2019) demonstrated for *N. pumilio* that some proteins related to a high-temperature defensive response become activated at temperatures of more than 20 °C. This suggests that seedlings may suffer a certain level of stress around this temperature.

Infection by *H. decorticans* is more severe when soil moisture is low. This is not due to previous drought stress, as observed in treatment yr1dry->Pwet and yr1dry->Pdry. These findings are consistent with previous research on young *Pinus sylvestris* trees inoculated by *Leptographium wingfieldii*, which showed that drought during infection is more important than drought that occurred earlier (Croisé *et al.* 2001). Our results indicate that in low soil moisture conditions, and probably negative water potentials, *H. decorticans* cause severe disease in seedlings. This is important because it suggests that drought may make Coihue seedlings more susceptible to the pathogen, and that *H. decorticans* may be more virulent in dry conditions. These combined factors create a synergistic effect that poses a greater threat to Coihue trees.

### Effects of pathogen on vascular tissues damage

The mycobiota in Coihue forests may play a secondary role in tree damage processes (Molina *et al.* 2020). However, the defense mechanisms of Coihue trees against diseases produced by pathogens are not fully understood. Evidence that supports this claim is the observation that the necrosis extension was much lower in the treatment exposed to dry condition in previous seasons and wet condition during the experiment (yr1dry->Pwet) than in the rest of the inoculated treatments. This suggests that trees were able to mount a defense against the disease when grown in favourable environmental conditions after being stressed by drought in previous seasons. These findings indicate that prolonged exposure to unfavourable conditions from previous seasons may trigger plant defenses against the disease caused by *H. decorticans*, particularly under favourable environmental conditions. Several studies demonstrate this fact, where it is verified that certain levels of drought induce the plant to activate defenses. For example, studies on *Arabidopsis* showed that certain water stress levels can provide resistance to *Pseudomonas syringae* infection by inducing specific marker genes and altering abscisic acid defense pathways (Gupta *et al.* 2016). In the *Vitis vinifera*, drought was found to increase gene expression related to plant defense (Cramer *et al.* 2007). Other studies on *Cajanus cajan* under stress due to *Fusarium udum* infection and salt stress showed that the same 11 genes were activated by the 2 stressors (Kumar *et al.* 2019).

From another perspective, the indirect effect of low moisture condition could favour the growth of mutualistic or competitor species of the pathogenic fungus. The humidity-dependent dynamic has not been studied for native *Nothofagus*, but it has been shown that certain antifungal endophytic *Streptomycetes* are present in *Nothofagus* species from northern Patagonia (Castillo *et al.* 2007). In this sense, if a low moisture content favours the development of this type of endophytes, the infection of pathogenic fungi such as *H. decorticans* could be reduced.

### Effects of moisture content and inoculation on growth

The response of Coihue to the variations in moisture conditions and presence or not of the pathogen is different according to the response variable considered. The height growth rate is not sensitive to the variations produced by moisture content and presence of pathogens. This response was also found in other studies related to moisture content in soil (Caselli *et al.* 2019). On the contrary, we found a direct relation between radial increase growth and moisture condition. The radial increase response to water restriction has been studied for tree species from Chile (Donoso *et al.* 2011; Peña-Rojas *et al.* 2017) and for several Mediterranean species (Peña-Rojas *et al.* 2004). In this sense, plants experiencing water stress showed a reduction in the rate of radial growth (Peña-Rojas *et al.* 2017).

Related to the effect of pathogens, there are not many studies on radial tree growth and the influence of fungi, most of the studies focus on root rot pathogens. Studies such as Eckhardt *et al.* (2004) indicate that infection of sapwood by *Leptographium* spp. results in occlusion, which reduces radial growth. However, based on the results of this experiment, it is not possible to differentiate between the negative effects of the pathogen and low moisture content on growth when both factors are combined. In seedlings inoculated with *H. decorticans* under wet conditions, the growth could be attributed to the cessation of the pathogenic activity by *H. decorticans* due to the normal water potentials in the plant, according to what was previously stated.

Regarding the differences we found between the provenances, this fact could be due to different cultivation methods and age rather than the seed provenances. Grossnickle and El-Kassaby (2016) found differences in the anatomy and performance of seedlings due to cultivation methods and planting sites. In addition, in *Austrocedrus chilensis* seedlings produced by different cultivation methods, Urretavizcaya *et al.* (2017) observed differences in growth and morphology that depended on cultivation method and site location. Related to seedling age, in general, seedlings show a period of rapid growth in the first year, followed by a slower rate of growth in the second year. Example of this is Rehfeldt’s (1979) studies, where the radial growth of Douglas-fir seedlings was significantly affected by age, with seedlings in their second year of growth having a 30 % greater radial growth than seedlings in their first year of growth. In this sense, the influence during the experiment of biotic and abiotic factors on Coihue seedling growth due to different cultivation methods or age is unknown.

## Conclusions

Our results confirmed that *H. decorticans* can act as a vascular stain pathogen on stressed Coihue plants (Supplementary material 3). The pathogen, in a plant exposed to low soil moisture or drought, affects the vascular system producing tissue necrosis and causing hydraulic failure, resulting in plant death. This capacity would be negatively affected in those plants exposed to previous drought seasons, where the plant’s defenses would be activated, thus reducing the possible action of the pathogen.

Future research focusing on these interactions should investigate the factors that accelerate mortality in Coihue forests under climate change scenarios that alter water and temperature cycles and impact trophic chains. It is essential to determine the environmental conditions that can favour or disrupt the natural dynamics of these forest systems.

It is expected that the predicted temperature increase due to climate change will bring frequent drought events (IPCC 2022). In this scenario, climatic conditions will be detrimental for Coihue in the presence of pathogens such as *H. decorticans*. Based on our results, Coihue may suffer increased mortality due to the combination of greater exposure to conditions of water deficit and *H. decorticans infection*.

## Supporting Information

The following additional information is available in the online version of this article.

Supplementary material 1: Monthly average soil temperature (°C) recorded during the experiment. Moisture content levels: dry (30–50 % of FC) and wet (70–90 % of FC).

Supplementary material 2: Trend lines of the partial increases in basal stem diameter as a function of the treatment moisture content for both provenances, regardless of the presence of the pathogen. Points represent the measured partial increments in each campaign.

Supplementary material 3: Detail of the healing callus at the end of the experiment, of a healthy plant inoculated only with agar (A). Detail of the necrosis along the stem without healing callus at the end of the experiment (B). Cross-section of the stem in sections showing the extent of necrosis within the sapwood (C).

plad068_suppl_Supplementary_Materials_S1

plad068_suppl_Supplementary_Materials_S2

plad068_suppl_Supplementary_Materials_S3

## Data Availability

The data underlying this article are available in [Zenodo], at https://doi.org/10.5281/zenodo.8384016.
