# Supplementary figures and images for: Effect of Huntiella decorticans and drought on Nothofagus dombeyi seedlings
Source: AoB Plants. 2023 Oct 10;15(5):plad068. doi: 10.1093/aobpla/plad068 (PMC10601059; doi:10.1093/aobpla/plad068)

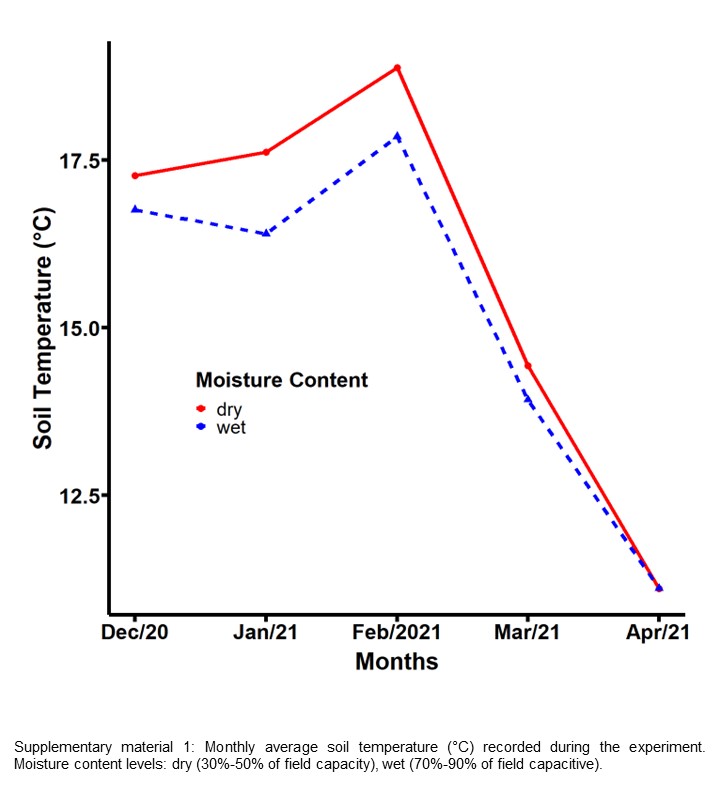

Supplement: plad068_suppl_Supplementary_Materials_S1 [file plad068_suppl_supplementary_materials_s1.jpeg]

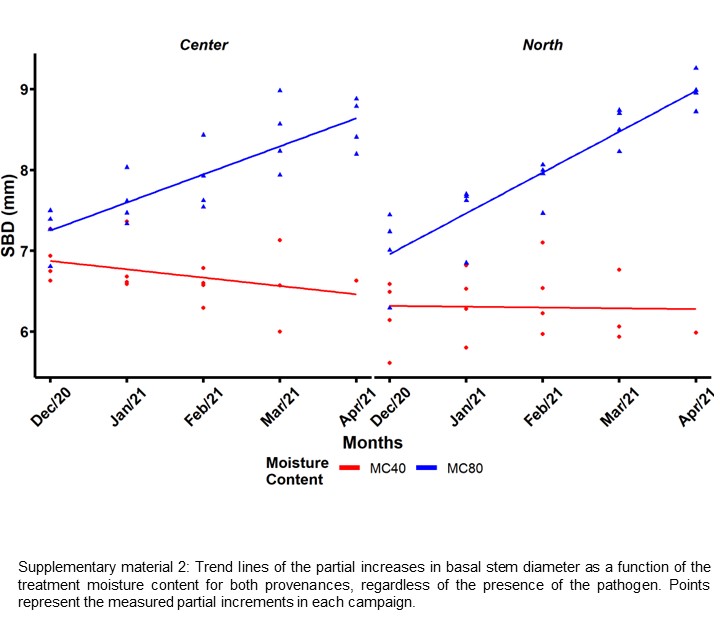

Supplement: plad068_suppl_Supplementary_Materials_S2 [file plad068_suppl_supplementary_materials_s2.jpeg]

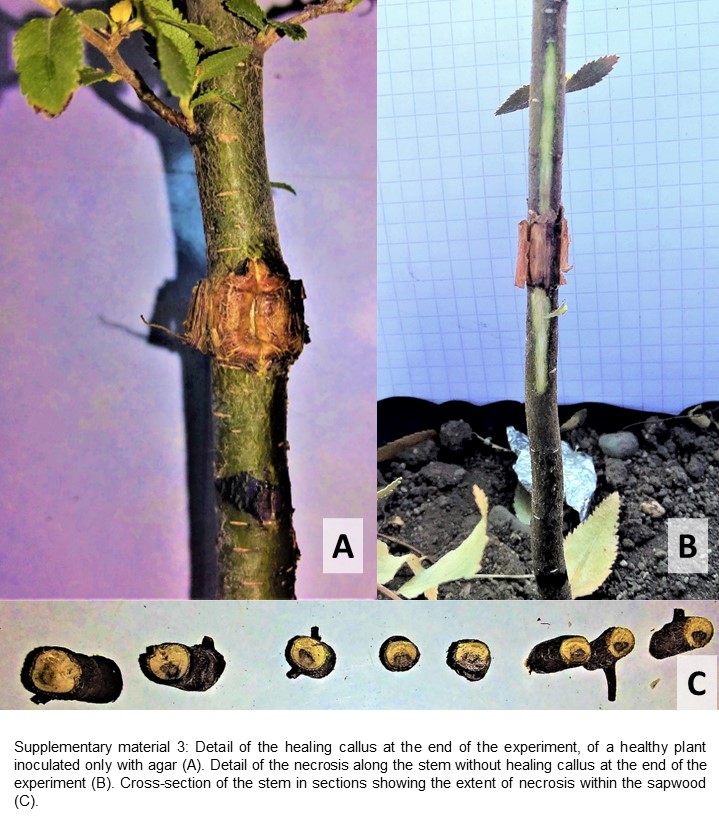

Supplement: plad068_suppl_Supplementary_Materials_S3 [file plad068_suppl_supplementary_materials_s3.jpeg]
